# Qualitative Exploration of the Potential for Adverse Events When Using an Online Peer Support Network for Mental Health: Cross-Sectional Survey

**DOI:** 10.2196/mental.8168

**Published:** 2017-10-30

**Authors:** Katherine Easton, Jacob Diggle, Mabel Ruethi-Davis, Megan Holmes, Darian Byron-Parker, Jessica Nuttall, Chris Blackmore

**Affiliations:** ^1^ Centre of Assistive Technology and Connected Healthcare University of Sheffield Sheffield United Kingdom; ^2^ Mind London United Kingdom; ^3^ The Medical School University of Sheffield Sheffield United Kingdom; ^4^ School of Health and Related Research University of Sheffield Sheffield United Kingdom

**Keywords:** digital technology, mental health, online peer support, adverse events, online survey

## Abstract

**Background:**

Online peer support networks are a growing area of mental health support for offering social connection, identity, and support. However, it has been reported that not all individuals have a positive experience on such networks. The potential for adverse events within a moderated online peer support network is a new area of research exploration.

**Objective:**

The objective of the study was to determine if use of an online moderated peer networks leads to adverse events for users.

**Methods:**

Four biannual online surveys (October 2014 to March 2016) were conducted by a large national UK mental health charity, with users of their online peer support network exploring personal safety, moderation, experiences on the site, and how the site could be improved. Data were analyzed using thematic analysis by 2 independent researchers using a priori themes: negative experiences of moderation, social exclusion, contagion, negative interactions with other users, online relationships, co-rumination and collusion, and other.

**Results:**

In total, 2353 survey responses were logged with 197 (8.37%) documenting an adverse event of negative experience. A dominant theme of negative experiences of moderation emerged (73/197, 37.1%) with evidence of social exclusion (50/197, 25.4%). Reading user posts was shown to be a cause of worry and distress for a few users, and analysis highlighted several instances of depressogenic and emotional contagion as well as some limited evidence of behavioral contagion (46/197, 23.4%). Very limited evidence of co-rumination (1/197, 0.5%) and no evidence of collusion were identified.

**Conclusions:**

Evidence of adverse events was identified at low levels in the sample of respondents, although we have no comparison data to indicate if levels are low compared with comparable platforms. Not all users of online peer support networks find them wholly beneficial. Research must explore what works for whom. The next stage of service development should consider which users may be likely to receive no benefit, or even deteriorate, as a result of using the service.

## Introduction

### Online Peer Support

Online peer support is an example of a technology-supported mental health self-management approach. Peer support has been defined as “people drawing on shared personal experience to provide knowledge, social interaction, emotional assistance, or practical help to each other, often in a way that is mutually beneficial” [1]. Peer support is often considered a helpful contribution to a wider mental health support and recovery plan. The approach works on the principles of using mutuality and reciprocity, which in turn facilitate the generation of social capital, known to be associated with well-being and resilience in mental health [2].

Evidence for the benefits of online peer support is mixed due in large part to the heterogeneous outcome measures adopted in research [3-8]. When using validated, clinical outcome measures based on a biomedical model to measure effectiveness, online peer support generally appears to have little effect [3,5,6]. When outcomes such as social connectedness, personal empowerment, and quality of life are assessed, research demonstrates evidence of benefits to users [4,7,8]. These are outcomes that are given a higher priority by people taking part in peer support [9].

### Adverse Events

The potential for adverse events when using peer support requires attention. Research has identified the presence of adverse events in online peer support including negative perceptions of moderation [8,10,11], emotional and behavioral contagion [11-15], negative debate [8,14], co-rumination [16] , collusion [13], and negative interactions with other users [8,11]. Other potentials for harm included unanswered posts and sharing of incorrect or misleading information about mental health [8]. The literature surrounding potential for harm on online peer networks is limited with a focus on depression. It is important to explore the user-perceived adverse events of using a national online peer support network for people with a range of mental health problems. In identifying the potential for adverse events on such platforms, the user experience may be improved, increasing safety and opportunity for beneficial outcomes.

## Methods

A qualitative analysis was conducted using a deductive framework approach [17] with data from 5 online service evaluation surveys posted on the Elefriends peer support platform sponsored by the UK mental health charity Mind from October 2013 to October 2016. Elefriends is a moderated online community with over 50,000 users. Moderation of the site is overseen by Ele handlers, who remove posts containing personal details, swearing, personal attacks, harassment, and potentially triggering content.

All open-ended questions on the online survey were screened for evidence of adverse events. Items included: What could Ele (the moderator) do to make you feel safer? Tell us about being an elefriend (user). What usually brings you to Elefriends? What effect has being an Elefriend had on you? Has being part of this community changed the way you access support? Has being part of this community encouraged you to try anything new? How do you feel about the moderation? Is there anything else you would like to tell us? How does Ele make you feel?

After investigating evidence of harm and negative outcomes of peer support in the literature, a framework was developed to classify the qualitative data. Initially, there were 7 broad categories: negative experiences of moderation, social exclusion, contagion, negative interactions with other users, online relationships, co-rumination and collusion, and other (Table 1). Responses to items on the surveys including those asking about personal safety, moderation, experiences on the site, and how the site could be improved were examined independently by 2 raters and verified by a third to identify evidence of the themes.

**Table 1:** Themes of adverse events and responses identified in user feedback.

Theme	n
Negative experiences of moderation or site management	73
Social exclusion	50
Contagion or trigger words	46
Negative interactions with other users	38
Online relationships	19
Co-rumination and collusion	1
Other	3

## Results

### Sample Characteristics

In total, 2353 survey responses were logged across the 5 surveys. Of the 1574 respondents, 79.01% were female (1258/1574) and 1.08% (17/1574) identified as transgender or other. A total of 29.86% (470/1574) of the sample was aged 18 to 25 years, and 28.84% (454/1574) was aged 25 to 35 years. Users were experiencing depression (384/1574, 24.39%), anxiety (157/1574, 9.97%), or both (587/1574, 37.29%); in addition, 10.42% (164/1574) reported personality disorders and 5.97% (94/1574) reported a diagnosis of posttraumatic stress disorder. A total of 8.37% (197/2353) of responses documented an adverse event of negative experience with 1.15% (27/2353) identifying multiple adverse events, which have been listed under multiple themes.

### Themes

The dominant theme of adverse events was moderation, commonly relating to censoring (73/2353).

...getting a post taken off for talking about what’s been upsetting me makes the matter even worse for myself...I feel even more alone than before...it just adds an extra weight on top of my bad time.

Response #6, Question 23, October 2015

Some users of the site experienced social exclusion or found it difficult to identify with others (50/2353).

...I find that my posts often receive little to no response, that in turn causes feelings of being ignored and invisible or unimportant and unpopular...even something as small as somebody not clicking the ‘hear you or thinking of you’ buttons can be so discouraging and disheartening; it feels as though nobody is reading our posts and instead looking for the more popular, regular posters and users.

Response #68, Question 20, May 2015

Evidence of distress, depressogenic, emotional, or behavioral contagion was also reported (46/2353).

I don’t like seeing people making suicide threats on the page. It’s scary for them and frustrating for me as I feel I am putting a lot of energy into positive thinking and that brings me down.

Response #82, Question 17, February 2015

I had to stop for a while because I felt mentally too fragile to listen to other people's struggles. Somehow their depression exacerbated my own.

Response #103, Question 18, February 2015

No evidence of collusion was found. Very limited evidence was found of co-rumination (1/2353).

I also get worried that sometimes it’s an echo chamber...It can sometimes reinforce negative or unhelpful behaviour...I don’t know if I’d have wanted to be an Elefriend when I was really bad. Surrounding myself with other people who agreed with me that life was basically awful may not have been very helpful.

Response #46, Question 18, February 2015

## Discussion

### Principal Findings

Evidence of adverse events was identified but appears to be at low levels in the sample of respondents. Moderation, social exclusion, and emotional contagion were identified in user responses although there is little data to indicate whether the rates we identified are comparable to other available platforms. People taking part in online peer support should feel able to express themselves, but the adverse events of removing posts must be weighed against the risks of leaving them in place, such as the increased contagion and collusion observed on unmoderated platforms [18,19]. Evidence suggests that depression, emotions, and behaviors can pass from one person to another [20]. However, we do not yet know at what rate such interactions need to occur for contagion to have a negative impact on other users. In terms of moderation, it is clear that a balance must be struck between reducing risk of contagion and allowing users in distress to express themselves and receive peer support.

Co-rumination and collusion can reinforce or encourage negative thoughts, perceptions, and behaviors, and this can facilitate contagion [19,20]. No evidence of collusion and very limited evidence of co-rumination was found in our study. This, as well as the lack of evidence of online stalking and sharing of health misinformation, can likely be attributed to the thorough nature of moderation on Elefriends.

Social exclusion and isolation are associated with depressive symptoms and have a detrimental impact on mental health [21,22]. Posts on Elefriends receive an average of 2.5 comments. Innovative ways to encourage users to interact with other posts and offer support, as well as writing their own posts, could be trialed.

Findings should be considered in light of the small self-selected sample and the sample characteristic. Individuals experiencing symptoms of depression or anxiety such as negative thought cycles are prone to catastrophizing, overgeneralization, and discounting positives, which may have influenced reporting of adverse events [23].

### Conclusions

Examination of online peer support user feedback indicates that adverse events are uncommon compared with concerns relating to moderation and social exclusion. Not all users of online peer support networks find them wholly beneficial. Research must explore what works for whom. The next stage of service development should consider which users may be likely to receive no benefit, or even deteriorate, as a result of using the service.
